# The Posterior Insula Shows Disrupted Brain Functional Connectivity in Female Migraineurs Without Aura Based on Brainnetome Atlas

**DOI:** 10.1038/s41598-017-17069-8

**Published:** 2017-12-04

**Authors:** Jilei Zhang, Jingjing Su, Mengxing Wang, Ying Zhao, Qi-Ting Zhang, Qian Yao, Haifeng Lu, Hui Zhang, Ge-Fei Li, Yi-Lan Wu, Yi-Sheng Liu, Feng-Di Liu, Mei-Ting Zhuang, Yan-Hui Shi, Tian-Yu Hou, Rong Zhao, Yuan Qiao, Jianqi Li, Jian-Ren Liu, Xiaoxia Du

**Affiliations:** 1 Shanghai Key Laboratory of Magnetic Resonance and Department of Physics, School of Physics and Materials Science, East China Normal University, Shanghai, 200062 China; 2 Department of Neurology and Jiuyuan Municipal Stroke Center, Shanghai Ninth People’s Hospital, Shanghai Jiao Tong University School of Medicine, Shanghai, 200011 China; 3 Clinical Research Center, Shanghai Jiao Tong University School of Medicine, Shanghai, 200011 China

## Abstract

Long-term headache attacks may cause human brain network reorganization in patients with migraine. In the current study, we calculated the topologic properties of functional networks based on the Brainnetome atlas using graph theory analysis in 29 female migraineurs without aura (MWoA) and in 29 female age-matched healthy controls. Compared with controls, female MWoA exhibited that the network properties altered, and the nodal centralities decreased/increased in some brain areas. In particular, the right posterior insula and the left medial superior occipital gyrus of patients exhibited significantly decreased nodal centrality compared with healthy controls. Furthermore, female MWoA exhibited a disrupted functional network, and notably, the two sub-regions of the right posterior insula exhibited decreased functional connectivity with many other brain regions. The topological metrics of functional networks in female MWoA included alterations in the nodal centrality of brain regions and disrupted connections between pair regions primarily involved in the discrimination of sensory features of pain, pain modulation or processing and sensory integration processing. In addition, the posterior insula decreased the nodal centrality, and exhibited disrupted connectivity with many other brain areas in female migraineurs, which suggests that the posterior insula plays an important role in female migraine pathology.

## Introduction

Migraines represent a recurrent and disabling neurological disorder associated with a combination of nausea, vomiting, tiredness, phonophobia, and photophobia, etc^1^. Migraines have become a major public health concern and plague nearly one in nine adults worldwide^2^. The prevalence of female migraineurs is two to three times that of male migraineurs, and females tend to require a longer time to recover and experience a longer attack duration, greater disability, and increased risk of headache recurrence^3,4^. Many neuroimaging studies have employed functional magnetic resonance imaging (fMRI), which provides a feasible, efficient and noninvasive tool to investigate the pathophysiological mechanisms of migraine. fMRI has revealed various dysfunctional brain areas in migraine mainly distributed in the the salience network, default mode network, the sensorimotor network, and the executive control network^5–15^. Our group recently investigated the abnormal patterns of functional networks of migraine compared with controls and found that migraineurs without aura (MWoA) showed the default mode network and sensorimotor network dysfunction^12,15^. However, these studies primarily focused on local dysfunction of brain regions or single intrinsic brain networks and neglected the information of connectome-scale aspect.

The human brain is a complex system that can be modeled as an extremely complex network consisting of edges (connections between pair regions) and nodes (brain areas)^16–18^. Graph theory analysis was a useful method for researching the functional integration and segregation patterns of brain networks and to investigate both global and local metrics. Previous brain network studies based on the Automated Anatomic Labeling (AAL) atlas have suggested that migraineurs exhibit alterations of topological properties and long-term headache attacks may cause human brain network reorganization in migraineurs^19–22^. Liu *et al*. found gender-related changes in the topological properties of functional networks and suggested that the networks of females may be more vulnerable to migraine^19^, finding hierarchical changes of structural and functional networks in female migraineurs^20^.

Previous research have shown that females are more likely to have migraine than males^4^, and the functional networks of females may be more vulnerable to migraine^19^, suggesting that the function and structure of the insula may be altered in migraine, especially in female migraineurs^23,24^. The female migraineurs showed a lack of thinning in the insula with age in contrast to healthy subjects^24^. In addition, female migraineurs exhibited increased cortical thickness in the posterior insular cortices compared with male patients with migraine and healthy controls^23^. The insula has been found to exhibit reductions in gray matter volume^25^, and function abnormal activation during the processing of pain-related words^26^, and aberrant connection with other regions^10,27–30^, which indicate that the insula structure and function are altered in migraine^31^. In fact, the insula was proposed as a “hub of activity” in migraine, as it processes and relays afferent inputs from frontal, temporal and parietal regions^31^. There are several functionally distinct areas within the insula: the posterior insula is mainly involved in somatosensory processes, the anterior insula in cognitive function, and the inferior insula in chemical sensory and social-emotional function^32–34^. A previous study using a region-of-interest analysis and task-free fMRI reported that the anterior insula increased intrinsic connectivity in migraine without aura^29^. The dorsal posterior insular cortex has been demonstrated to play a fundamental role in human pain^35^. However, the insula has not been reported to be abnormal in previous graph theory studies based on the AAL atlas^19–22^, which may be due to its coarse sub-regions. Notably, the AAL atlas was acquired according to the anatomical sulcal information of brain of a single person, and it may be not suitable for group-level analysis because of individual differences in anatomical structure^36,37^. Recently, a more precise atlas, the Brainnetome atlas, with 36 subcortical subregions and 210 cortical was designed based on 40 healthy adults using noninvasive multimodal neuroimaging techniques and is freely available for download by researchers^38^. More importantly, the insula is divided into 12 subregions in the Brainnetome atlas. Thus, we think that the Brainnetome atlas could detect the local abnormal or the disrupted functional connectivity of the sub-regions of insula in migraineurs, and the functional changes of insula may be involved in its structural alterations.

Hence, graph theory analysis based on the Brainnetome atlas was used to explore and investigate the topological organization of the functional networks of female MWoA, and we compared the differences in topological organizations of networks constructed by the AAL atlas and the Brainnetome atlas. Subsequently, the volumetric analysis in brain regions with nodal centrality alterations were used to investigate the corresponding structural changes. We hypothesized that the topological properties of functional networks, the functional connectivity patterns and the volume of the insular sub-regions would be altered in female MWoA.

## Methods

The Independent Ethics Committee of Shanghai Ninth People’s Hospital (Project No. [2016]01) and East China Normal University Committee on Human Research (Project No. HR2016/03022) approved the current study. All subjects were asked to provide written informed consent, which was approved by the committee. All methods were carried out in accordance with the principles outlined in the Declaration of Helsinki, including any relevant details.

### Subjects

Twenty-nine female MWoA (age ± SD, 40.1 ± 11.4) were recruited from among outpatients of the Department of Neurology at Shanghai Ninth People’s Hospital. According to the International Classification of Headache Disorders (ICHD-III beta, 2013), the headache patients were diagnosed as MWoA by a neurologist. MWoA were excluded from our study according to the following criteria: 1) patients who suffered a headache attack during 48 h before MRI scans; 2) patients who suffered from a migraine attack or who felt discomfort during the MRI scans; and 3) patients who were taking preventive medicines or with a chronic migraine. Twenty-nine female age-matched healthy controls (age ± SD, 39.8 ± 11.2) were recruited. The control subjects had not suffered headaches in the past year, and their immediate family members had not been diagnosed with migraines. According to structured interviews and clinical examinations, all participants had not suffered any neurological or psychiatric diseases, were free of substance abuse, and were right handed. The clinical data and demographics of the patients and controls are presented in Table 1.

**Table 1:** Clinical scores and demographics of the female MWoA and healthy controls.

	MWoA (29 female)	Healthy controls (29 female)
Age (years)	40.1 ± 11.4	39.8 ± 11.2
Disease duration (years)	9.4 ± 7.4	—
Attack duration (hours)	22.7 ± 22.1	—
Attack frequency (times/months)	3.3 ± 2.9	—
VAS	6.8 ± 2.0	—
MIDAS	15.6 ± 16.7	—
HIT-6	63.4 ± 10.3	—

MWoA: migraineurs without aura; HIT-6: Headache Impact Test; MIDAS: Migraine Disability Assessment Scale; VAS: visual analogue scale; -: no data.

### MRI acquisition

T_1_-weighted data with high-resolution and resting-state fMRI data were obtained from a 3 T Siemens scanner (Trio Tim, Germany) with a 12-channel head coil. To minimize head motion, custom-fit foam pads were used to fix the head of subjects. T_1_-weighted data with high-resolution were obtained using a 3-dimensional magnetization-prepared rapid-acquisition gradient-echo pulse sequence with the following parameters: 192 slices, matrix size = 256 × 256, field of view = 256 × 256 mm^2^, echo time = 2.34 ms, repetition time = 2530 ms, inversion time = 1100 ms, and slice thickness = 1 mm. The fMRI images were acquired using a T_2_*-weighted gradient-echo echo-planar imaging pulse sequence, with the following parameters: 210 volumes, transverse orientation, matrix size = 64 × 64, field of view = 220 × 220 mm^2^, echo time = 30 ms, repetition time = 2000 ms, 33 slices, 25% Dist factor, slice thickness = 3.5 mm, and flip angle = 90°. The patients and controls were instructed to remain still, close their eyes, and relax during the fMRI scan.

### Data preprocessing

Resting-state fMRI images preprocessing was performed with SPM 12 software (Statistical Parametric Mapping; http://www.fil.ion.ucl.ac.uk/spm/). The main processes were as follows: 1) To ensure that the participants adapted to the scanner noise and to avoid scanner instability, the first ten volumes were removed from the fMRI images. 2) Slice timing was conducted to correct the time delay of intra-volume acquisition. 3) Using rigid body (six parameters) spatial transformation, the fMRI data were realigned to the first volume. 4) The T1-weighted images were coregistered to the mean functional images. 5) The T_1_-weighted data were segmented into white matter and gray matter, and the forward deformation fields and the inverse deformation fields were generated using the “New Segment” method. 6) Spatial normalization was performed to register the functional images to the MNI space, and images were resampled to 3 mm × 3 mm × 3 mm. 7) The normalized images were spatially smoothed with a 4-mm full-width half-maximum (FWHM) Gaussian filter. 8) The linear trend of the smoothed images was removed, and the signals of the cerebrospinal fluid, the white matter and six parameters of head motion were regressed out as covariates. 8) To reduce the influence of high-frequency respiratory and cardiac noise and low-frequency drift, the data were temporally bandpass filtered (0.01 < f < 0.1 Hz).

In the current study, none of he subjects were excluded, as the maximum rotation movement was <2° and the translation movement was < 2 mm for all subjects. We compared head motion parameters in any direction between the control and migraine groups using a nonparametric permutation test (50000 permutations) and we did not find any significant difference (pitch, p = 0.72; roll, p = 0.57; yaw, p = 0.72; x, p = 0.37; y, p = 0.30; z, p = 0.19).

### Network construction and analysis based on the Brainnetome atlas

We constructed the functional network with the graph theoretical network analysis (GRETNA) toolbox^39^. The human Brainnetome atlas^38^ was employed to define the brain nodes, and each brain area represents a node of the network (Supplementary Table S1). The average time series was generated from the human Brainnetome atlas. Finally, these time series were used to calculate the correlation coefficient using Pearson correlation, and 246 × 246 correlation matrices for participants were generated for graph theory analysis.

To address the differences in the number of edges within subjects, a range sparsity threshold S (0.10 ≤ S ≤ 0.30, interval 0.01) were applied to all network matrix. We defined sparsity as the actual number of edges of a specific network divided by the maximum possible number of edges^40^. The range of S which was chosen to ensure that each thresholded network had small-worldness (σ > 1.1) and the mean degree of all brain areas for each thresholded network was greater than 2 × log (246), and was consistent with previous studies^41,42^. For functional network at each sparsity threshold, a binary matrix was generated from the correlation matrix if the positive correlation values between two brain regions exceeded a given sparsity threshold, and then, both the global and nodal measures of all networks were calculated.

We calculated the small-world parameters, network efficiency and nodal centrality as follows: (1) small-world parameters: characteristic path length (L_p_), clustering coefficient (C_p_), normalized characteristic path length (λ), normalized clustering coefficient (γ), and small-worldness (σ); (2) network efficiency: global efficiency (E_glob_) and local efficiency (E_loc_); (3) nodal centrality: nodal betweenness, efficiency and degree^42–44^. The definitions of network measures are presented in Supplementary Table S2. Furthermore, the areas under the curve (AUC) for each network metric (Y) were calculated as:

$${Y}^{AUC}=\,\frac{1}{2}\,\sum _{K=1}^{N-1}\,({Y}_{{S}_{k}}+{Y}_{{S}_{k+1}})\,\times {\rm{\Delta }}S$$

**Figure 1:**
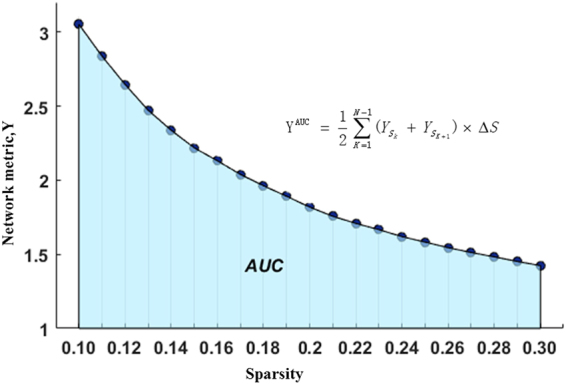
The AUC for a network metric Y, which was calculated over the sparsity threshold range of S_1_ to S_n_ with an interval of ΔS (S_1_ = 0.10, S_n_ = 0.30, and ΔS = 0.01).

In the current study, S_1_ = 0.10, S_n_ = 0.30, and ΔS = 0.01 (Fig. 1). The AUC metric provides a summarized measure for the global and local parameters of functional networks rather than a single threshold selection and is sensitive to detecting topological changes in neurological disorders, as previously reported^41,45^.

### Network construction and analysis based on the AAL atlas

We also constructed functional networks based on Automated Anatomical Labeling^37^ and calculated the global metrics (L_p_, C_p_, λ, γ, σ, E_glob_, E_loc_) and nodal centrality (the nodal betweenness, efficiency and degree) according to the above methods.

### Statistical analysis

We compared the AUC of each network metric, including small-world properties (Lp, Cp, λ, γ, σ), network efficiency (E_glob_, E_loc_) and nodal characteristics (nodal betweenness, efficiency and degree), between female MWoA and healthy controls using nonparametric permutation tests. First, the difference in the average value of each metric between groups was calculated. Then, we randomly reassigned all the values of each metric into two groups and recalculated the difference in the average value of each metric between the two randomized groups. Finally, we repeated the randomization procedure 50,000 times to obtain probability distributions. We used false discovery rate (FDR) correction to address the problem of multiple comparisons(P < 0.05).

Moreover, the nonparametric permutation test (50000 permutations) was used to localize the functional connectivity that significantly changed in the large-scale brain networks of female MWoA compared with healthy controls using the NBS connectome toolbox (version 1.2)^46^. FDR correction was used to address the problem of multiple comparisons (P < 0.01).

### Volumetric analysis

The inverse deformation field were generated from previous preprocessing procedure. Then, the Brainnetome atlas were projected onto the individual space by applying the inverse deformation field, which would construct the individual Briannetome atlas. We calculated the subject-specific volume of brain regions with nodal centrality changes (FDR corrected), and then computed the ratio of brain region volume/total gray matter volume to eliminate the differences of total gray matter volume of subjects. Finally, the corrected volumetric differences were compared between migraine patients and controls using the nonparametric permutation test (50000 permutations).

### Correlation analysis

The values of significant alterations in the global and nodal characteristics were correlated with the clinical data of migraineurs using Pearson correlation analysis, including attack frequency, attack duration, and disease duration, as well as the Headache Impact Test (HIT-6), Migraine Disability Assessment Scale (MIDAS) scores and visual analogue scale (VAS). Significant correlations were determined based on uncorrected p-values(p < 0.05).

## Results

### Global and reginal topological organizations of the functional networks based on the Brainnetome atlas

**Figure 2:**
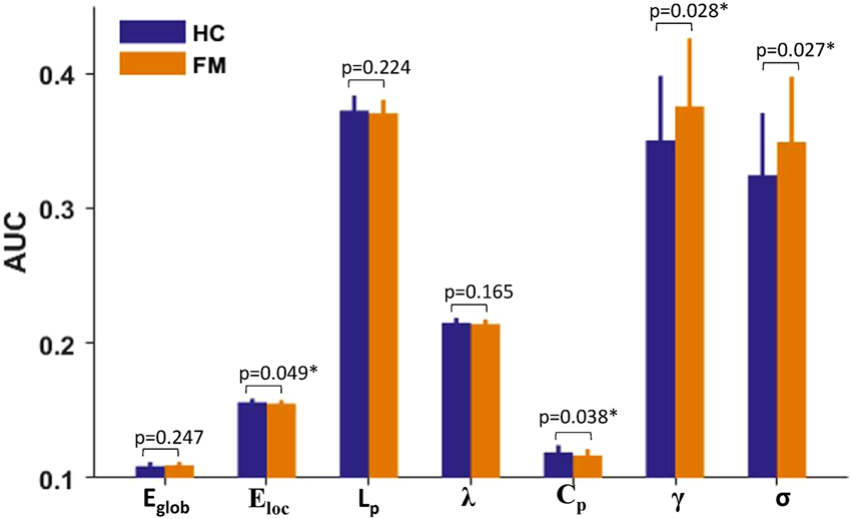
The differences in the topological organizations of brain networks based on the Brainnetome atlas between female MWoA and healthy controls. Error bars denote standard deviations. *Significant difference between two groups (P < 0.05, uncorrected, 50000 permutations test); HC: healthy controls; FM: female MWoA; E_glob_: global efficiency; E_loc_: local efficiency; L_p_: characteristic path length; λ: normalized characteristic path length; C_p_: clustering coefficient; γ: normalized clustering coefficient; σ: small-worldness.

The functional networks of female MWoA and healthy controls had higher clustering coefficients (C_p_) and similar characteristic path lengths (L_p_) than the random networks, which indicated that both female MWoA and healthy controls exhibited typical small-world topology properties. The female MWoA exhibited significantly lower C_p_ and E_loc_ values and higher γ and σ values (P < 0.05, uncorrected, nonparametric permutation test) than the control group. There were no differences in L_p_, λ, and E_glob_ between the healthy controls and female MWoA. The details are presented in Fig. 2.

**Figure 3:**
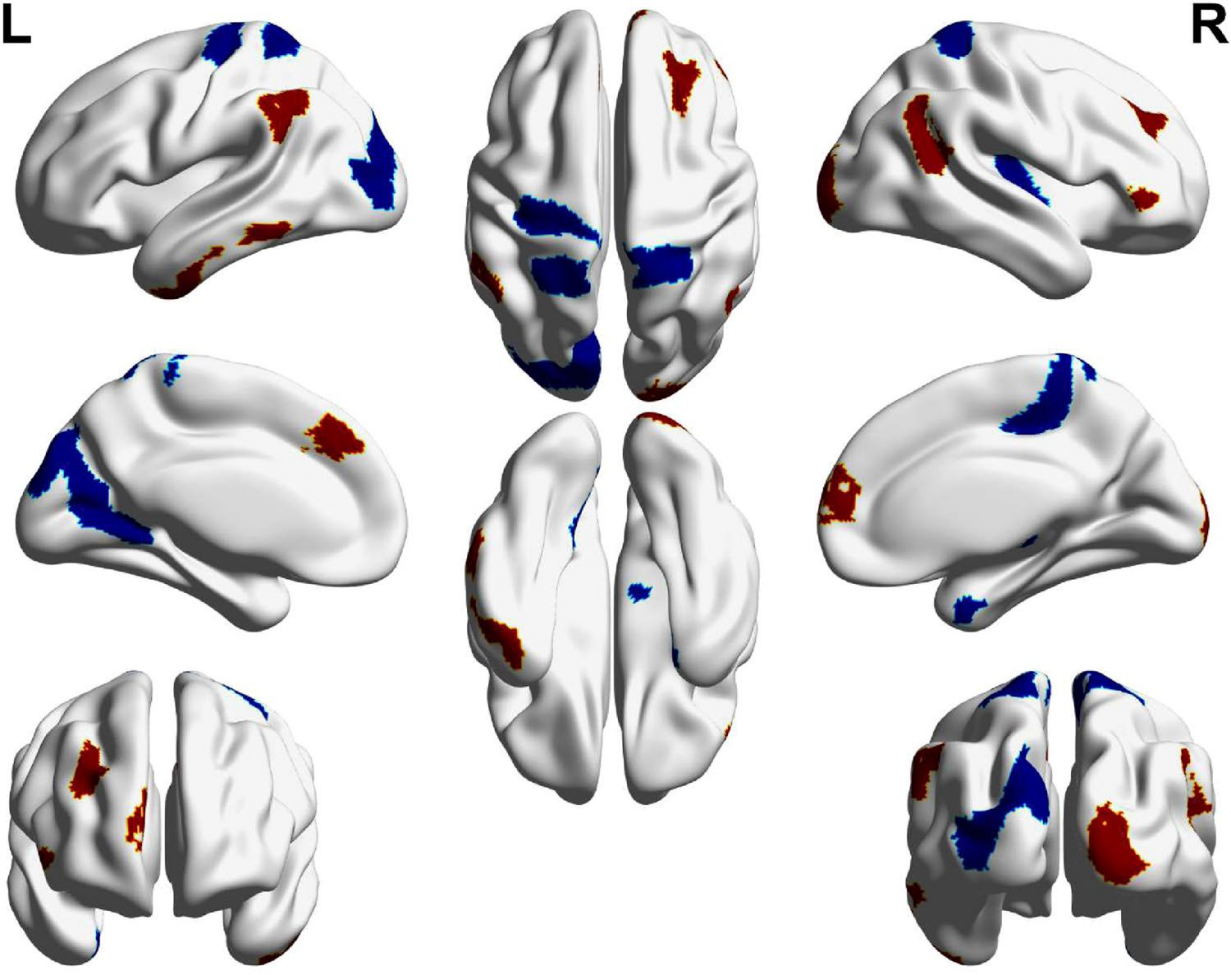
Subregions of the brain within the Brainnetome atlas showing abnormal nodal centrality in female MWoA compared with healthy controls. Compared with healthy controls, the MWoA showed decreased (blue color) nodal centralities in the precentral gyrus (BA4), the right parahippocampal gyrus, the left paracentral lobule, the bilateral superior parietal lobule (BA7), the right posterior insula, the left occipital cortex and the sensory thalamus. The migraineurs showed increased (red color) nodal centralities in the PFC (left BA9, right BA9/10/45/47), the bilateral inferior parietal lobes (left BA40 and right BA39), the left inferior temporal gyrus (BA20/37) and the right occipital polar cortex.

We report the brain regions exhibiting significant differences between groups in at least one nodal metric. The right posterior insula (hypergranular insula) and the left medial superior occipital gyrus of female MWoA had significantly decreased nodal centrality compared with healthy controls (P < 0.05, FDR corrected, nonparametric permutation test). We also report the brain regions exhibiting significant differences between groups in uncorrected level (P < 0.005, uncorrected, nonparametric permutation test). Compared with healthy controls, female MWoA exhibited decreased nodal centralities in the left precentral gyrus (BA 4, upper limb region), the right paracentral lobule, the right parahippocampal gyrus, the bilateral superior parietal lobule (postcentral BA7), the right posterior insula (hypergranular insula), the left occipital cortex (ventromedial parietooccipital sulcus, middle and medial-superior occipital gyrus) and the right thalamus (sensory thalamus) (P < 0.005, nonparametric permutation test, uncorrected). The female MWoA exhibited increased nodal centralities in the bilateral superior frontal gyrus (medial BA 9 and medial BA 10), the right middle frontal gyrus (dorsal BA 9/46), the right inferior frontal gyrus (rostral BA 45), the left inferior temporal gyrus (caudolateral and rostral BA 20, ventrolateral BA 37), the bilateral inferior parietal lobule (rostroventral BA 39 and caudal BA 40), and the right occipital polar cortex (P < 0.005, nonparametric permutation test, uncorrected). The details are presented in Fig. 3 and Table 2.

**Table 2:** Subregions of the brain within the Brainnetome atlas showing abnormal nodal centrality in female MWoA versus healthy controls.

Brain regions	Anatomical Structure	Nodal betweenness _Mean (SD)_			Nodal efficiency _Mean (SD)_			Nodal degree _Mean (SD)_		
		FMWoA	HC	P value	FMWoA	HC	P value	FMWoA	HC	P value
Nodal centrality increases in migraineurs										
SFG_L_7_6	medial BA 9	33.59(28.25)	21.87(29.02)	0.065	**0.114(0.011)**	**0.103(0.015)**	**0.002**	**10.62(3.92)**	**7.71(4.12)**	**0.004**
SFG_R_7_7	medial BA 10	30.51(26.12)	28.41(24.04)	0.373	0.117(0.013)	0.108(0.014)	0.008	**12.16(4.68)**	**8.60(4.47)**	**0.002**
MFG_R_7_1	dorsal BA 9/46	26.41(20.59)	22.50(20.24)	0.233	**0.113(0.010)**	**0.105(0.013)**	**0.004**	10.27(3.49)	7.69(3.76)	0.005
IFG_R_6_4	rostral BA 45	24.40(20.23)	26.26(27.49)	0.385	0.113(0.011)	0.106(0.010)	0.008	**10.08(4.11)**	**7.47(3.25)**	**0.004**
ITG_L_7_3	rostral BA 20	22.81(25.00)	11.81(14.15)	0.020	**0.106(0.012)**	**0.093(0.021)**	**0.003**	7.78(3.91)	5.44(3.6)	0.011
ITG_L_7_4	ventrolateral BA 37	24.61(19.20)	25.17(33.28)	0.472	0.110(0.015)	0.098(0.016)	0.008	**9.72(4.14)**	**6.75(4.14)**	**0.004**
ITG_L_7_6	caudolateral BA 20	20.76(18.02)	13.08(10.56)	0.026	**0.107(0.016)**	**0.094(0.022)**	**0.004**	**8.71(4.87)**	**5.61(3.76)**	**0.004**
IPL_L_6_4	caudal BA 40 (PFm)	24.72(26.51)	13.56(14.60)	0.023	0.108(0.015)	0.098(0.016)	0.006	**9.05(4.39)**	**5.84(3.02)**	**0.001**
IPL_R_6_5	rostroventral BA 39	25.54(16.15)	20.20(13.37)	0.087	**0.116(0.011)**	**0.107(0.011)**	**0.003**	**11.57(3.75)**	**8.41(3.88)**	**0.002**
LOcC _R_4_3	occipital polar cortex	**17.25(19.99)**	**7.29(6.97)**	**0.004**	0.106(0.019)	0.102(0.023)	0.238	8.83(5.90)	7.63(4.69)	0.196
Nodal centrality decreases in migraineurs										
PrG_L_6_3	BA 4(upper limb region)	22.14(17.80)	24.16(31.45)	0.407	**0.114(0.013)**	**0.124(0.010)**	**0.002**	**11.13(4.95)**	**14.93(3.92)**	**0.001**
PCL_R_2_1	BA 1/2/3 (lower limb region)	42.84(33.11)	46.11(37.82)	0.367	**0.120(0.010)**	**0.129(0.011)**	**0.001**	**12.85(4.01)**	**16.76(4.91)**	**0.001**
PhG_R_6_5	temporal agranular insular cortex	**2.97(5.00)**	**12.02(17.40)**	**0.004**	0.053(0.040)	0.075(0.039)	0.016	1.94(2.72)	4.02(3.95)	0.010
SPL_L_5_4	postcentral BA 7	19.87(16.70)	19.73(13.75)	0.485	**0.113(0.013)**	**0.122(0.009)**	**0.002**	**11.08(4.67)**	**14.34(3.78)**	**0.003**
SPL_R_5_4	postcentral BA 7	18.49(18.30)	20.62(18.84)	0.331	**0.112(0.013)**	**0.121(0.010)**	**0.002**	**10.28(4.81)**	**13.81(4.02)**	**0.002**
INS_R_6_1	hypergranular insula	17.67(18.53)	30.01(28.66)	0.013	**0.109(0.016)**	**0.121(0.011)**	**0.001**	**9.36(5.05)**	**13.74(4.24)**	0.000*
MVOcC _L_5_5	ventromedial parietooccipital sulcus	33.99(26.31)	31.52(21.78)	0.350	**0.118(0.012)**	**0.126(0.009)**	**0.004**	**12.61(4.59)**	**15.76(3.51)**	**0.002**
LOcC_L_4_1	middle occipital gyrus	9.15(9.67)	13.73(12.37)	0.062	**0.105(0.012)**	**0.115(0.014)**	**0.003**	**8.05(3.74)**	**11.60(5.29)**	**0.002**
LOcC _L_2_1	medial superior occipital gyrus	12.82(11.10)	17.16(14.17)	0.103	**0.110(0.011)**	**0.120(0.010)**	**0.000**	**9.80(3.68)**	**13.54(3.87)**	0.000*
Tha_R_8_3	sensory thalamus	**11.57(12.32)**	**32.20(58.01)**	**0.003**	0.105(0.014)	0.113(0.011)	0.007	**7.65(4.29)**	**10.55(3.97)**	**0.004**

Subregions of the brain were considered abnormal in female MWoA if they exhibited significant between-group differences (50000 permutations, P < 0.005 shown in bold font) in at least one of the three nodal centralities. “*”Indicates brain regions that are significant after multiple comparison correction (P < 0.05, nonparametric permutation test, FDR corrected). MWoA, migraineurs without aura; SFG, superior frontal gyrus; MFG, middle frontal gyrus; IFG, inferior frontal gyrus; ITG, inferior frontal gyrus; IPL, inferior parietal gyrus; LOcC, lateral occipital cortex; PhG, parahippocampal gyrus; PrG, precentral gyrus; PCL, paracentral lobule; SPL, superior parietal gyrus; INS, insula; MVOcC, medioventral occipital cortex; LOcC, lateral occipital cortex; Tha, thalamus; L, left; R, right; SD, standard deviation.

### Disrupted functional connectivity in female MWoA

**Figure 4:**
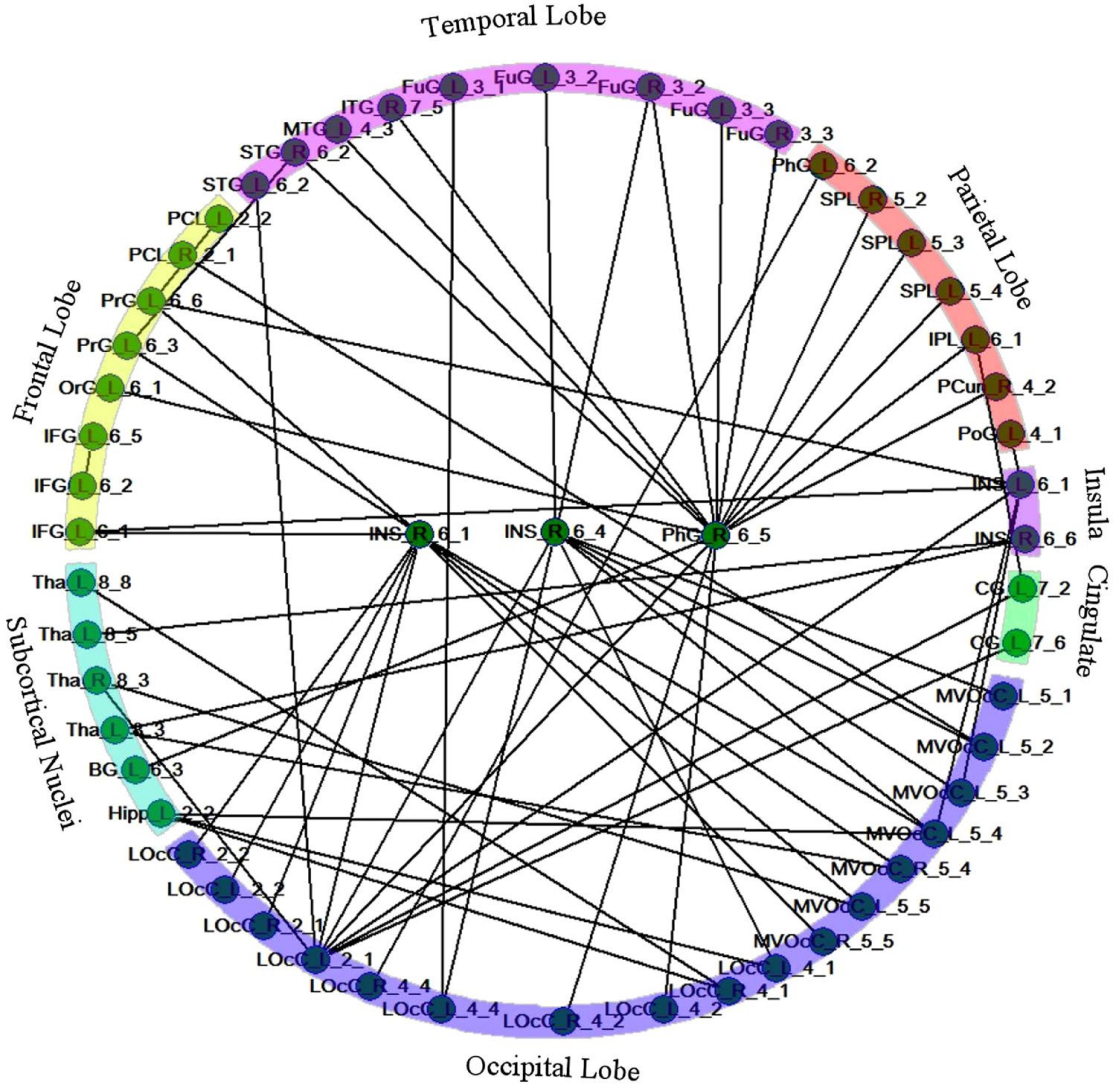
The disrupted connections within the brain network in female MWoA compared with healthy controls. The nodes were primarily located in the orbital frontal cortex, the sensory-motor cortex, the inferior frontal gyrus, the temporal lobe, the cingulate cortex, the posterior parietal lobe, the insular cortex, the occipital lobe and the subcortical nuclei. IFG: inferior frontal gyrus; OrG: orbital frontal gyrus; PrG: precentral gyrus; PCL: paracentral gyrus; STG: superior temporal gyrus; MTG: middle temporal gyrus; FuG: fusiform gyrus; PhG: parahippocampal gyrus; SPL: superior parietal lobule; IPL: inferior parietal gyrus; Pcun: precuneus; PoG: postcentral gyrus; CG: cingulate gyrus; MVOcC: medio ventral occipital cortex; LOcC: lateral occipital cortex; Hipp: hippocampus; BG: basal ganglia; Tha: thalamus.

We identified a disrupted or altered network with 61 connections within 54 sub-regions, and these connections were significantly decreased in female MWoA compared with controls (P < 0.01, FDR corrected, nonparametric permutation test) (Fig. 4). No increased connections were detected in MWoA. The sub-regions and disrupted connections were distributed in different lobes, including the orbital frontal cortex, the sensory-motor cortex, the inferior frontal gyrus, the cingulate cortex, the parietal lobe, the temporal lobe, the insular cortex, the occipital lobe and the subcortical nuclei (Fig. 4).

### Volumetric analysis

The volumetric analysis were performed in the posterior insula (INS_R_6_1) and the left medial superior occipital gyrus (LOcC _L_2_1) which exhibited the nodal centrality changes. The results exhibited that the posterior insula in female MWoA was significantly smaller than healthy controls (p = 0.049, uncorrected), and the left medial superior occipital gyrus showed no differences between groups. The details are presented in Table 3.

**Table 3:** The comparison of brain region volume between female MWoA patients and controls.

Brain region volume	Female MWoA (Mean ±SD)	HC (Mean ±SD)	p value
Total GMV	658.50 ± 41.18 ml	654.20 ± 42.93 ml	0.358
INS_R_6_1	2.243 ± 0.167 ml	2.289 ± 0.155 ml	0.138
INS_R_6_1/ Total GMV	0.341 ± 0.024%	0.351 ± 0.018%	0.049*
LOcC _L_2_1	3.452 ± 0.309 ml	3.494 ± 0.287 ml	0.296
LOcC _L_2_1/ Total GMV	0.525 ± 0.039%	0.535 ± 0.039%	0.153

The volume of posterior insula (INS_R_6_1) in female MWoA was significantly smaller than healthy controls, and the left medial superior occipital gyrus (LOcC _L_2_1) showed no differences between groups. “*”Indicates the p value was significant. MWoA, migraine without aura; HC, healthy controls; GMV, gray matter volume; INS, insula; LOcC, lateral occipital cortex; L, left; R, right; SD, standard deviation.

### Correlation with clinical scores

**Figure 5:**
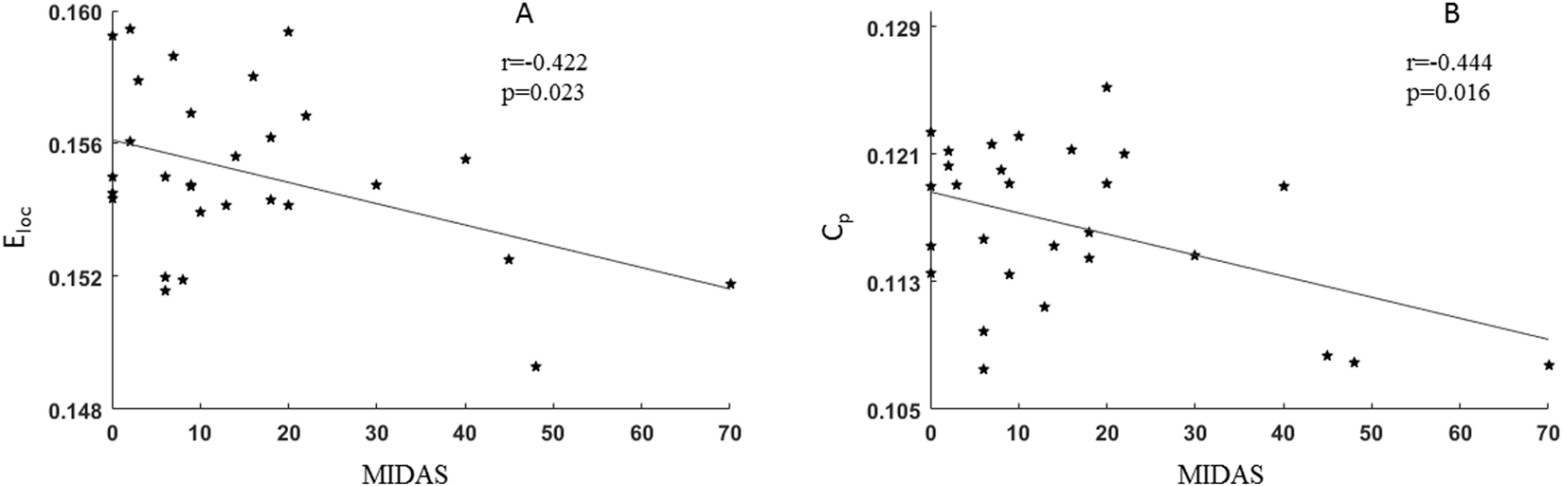
Scatterplots of global measures correlated with the MIDAS score. (**A**) The values of E_loc_ in female MWoA were negatively correlated with the MIDAS score (p = 0.023, r = −0.422), (**B**) The values of C_p_ in female MWoA were negatively correlated with the MIDAS score (p = 0.016, r = −0.444).

We found that E_loc_ (p = 0.023, r = −0.422, uncorrected) and C_p_ (p = 0.016, r = −0.444, uncorrected) in female MWoA were negatively correlated with the MIDAS score (Fig. 5). However, the other altered global and nodal characteristics were not significantly correlated with attack duration, disease duration, attack frequency, or the VAS and HIT-6 scores in the female MWoA.

### Global and regional topological organizations of the functional networks based on the AAL atlas

Upon analyzing the functional networks constructed by the AAL atlas, we only found that γ (P = 0.037) and σ (P = 0.028) of female MWoA were significantly increased compared with controls (Supplementary Figure S1). In addition, nodal centrality was significantly increased in the bilateral medial prefrontal cortex, the right orbital frontal cortex, the right posterior cingulate gyrus and the left inferior temporal gyrus and decreased in the left precentral gyrus, the left orbital frontal cortex, the left superior and middle occipital gyrus, the left postcentral gyrus, and the left pallidum (P < 0.005, nonparametric permutation test, uncorrected). The details are presented in Supplementary Figure S2 and Table S3.

## Discussion

In the current study, we calculated the small-world topology properties of networks based on the Brainnetome atlas and found that female MWoA exhibited significantly higher γ and σ values and lower C_p_ and E_loc_ values compared with controls (Fig. 2). The C_p_ and L_p_ of a network reveal the ability of functional segregation and integration, respectively^44^. More specifically, the brain’s functional segregation is the ability for specific processing within densely interconnected clusters of brain areas, and the brain’s integration is the ability to rapidly transfer and integrate specialized information from different brain areas^44^. The E_glob_ and E_loc_ of a graph measure the global and local efficiency of information, while λ, γ and σ reflect the small-worldness of a functional network^47^. The significantly lower C_p_ and E_loc_ values may suggest that the brain network of MWoA corresponded to decreased functional segregation that affected the local efficiency of information processing. In addition, C_p_ and E_loc_ in patients with migraine were negatively correlated with the MIDAS score (Fig. 5), which suggested that the topology properties of network alteration may be associated with the daily life of the patient and the pathophysiology of migraine. Conversely, the higher γ and σ in migraineurs may enhance the network’s small-worldness. The alterations in the small-world measures and network efficiency may be caused by alterations of the nodal centrality of specified brain areas that are involved in pain processing or modulation.

Nodal centrality, which is measured by nodal degree, efficiency and betweenness, is an important index for evaluating the importance of nodes within functional networks. The changes in nodal centrality for individual brain regions may affect the information processing efficiency and the functional integration or segregation of the functional network. In the current study, female migraineurs exhibited decreased nodal centralities in the precentral gyrus (BA4), the right parahippocampal gyrus, the left paracentral lobule, the bilateral superior parietal lobule (BA7), the right posterior insula, the left occipital cortex and the sensory thalamus compared with healthy controls (Fig. 3). The female migraineurs also showed increased nodal centralities in the PFC (left BA9, right BA9/10/45/47), the left inferior temporal gyrus (BA20/37), the bilateral inferior parietal lobes (left BA40 and right BA39) and the right occipital polar cortex(Fig. 3). These brain regions are involved in discrimination of sensory features of pain, and pain processing or modulation^13,31,48–51^. The prefrontal cortex has been shown to receive sensory information from pain-associated cortical areas (SI, ACC, SII/insula and thalamus) and plays an important role in cognitive modulation of pain^52^. The alterations of the prefrontal cortex in migraineurs are consistent with anatomical and functional changes reported in previous studies^12,53–56^. It has been proposed that the inferior and superior parietal lobules and the anterior cingulate cortex are involved in the spatial discrimination about sensory features of pain, and the bilateral dorsolateral prefrontal cortex and the insula are associated with intensity discrimination of sensory features of pain^48,49,51^. The temporal lobe is associated with multimodal sensory integration and has been demonstrated to be activated during migraine attacks and pain experiences^57,58^. A previous graph theory study of migraineurs found altered nodal efficiency of the prefrontal cortex, the insula and the paracentral lobule in structural networks based on the AAL atlas^21^. Thus, the nodal centrality alterations are in line with previous research and may affect the pathway of pain processing or modulation and even affect functional integration or segregation processing of the functional network in MWoA. Figure 3 shows that brain regions with increased and decreased nodal centrality coexist in female MWoA. We suppose that the brain regions with decreased nodal centrality may disrupt the pathway of pain processing or modulation, and conversely, the brain regions with increased nodal centrality may have compensatory functions or induce migraine patients to be more sensitive to pain^50,59^.

Furthermore, we identified a disrupted network with 61 connections within 54 nodes in the MWoA, and these nodes exhibited decreased connections distributed to different lobes, including the orbital frontal cortex, the sensory-motor cortex, the inferior frontal gyrus, the cingulate cortex, the posterior parietal lobe, the temporal lobe, the insular cortex, the occipital lobe and the subcortical nuclei (Fig. 4). In particular, the right posterior insula and the right parahippocampal gyrus exhibited disrupted connectivity with many of brain regions (Fig. 4). The subregions of the brain with disrupted connectivity in patients were involved in the discrimination of sensory features of pain (sensory-motor cortex, posterior insula and posterior parietal cortex), cognitive processing (orbitofrontal cortex and parahippocampal gyrus), pain modulation processing (thalamus, insula, anterior cingulate cortex, and prefrontal cortex), sensory integration processing (temporal lobe) and visual information processing (occipital lobe)^26,31,48,49,51,55,60–64^. The parahippocampal gyrus exhibited disrupted connectivity with the fusiform gyrus, the temporal lobe, the occipital lobe and the parietal lobe. These results are in line with previous research that also investigated abnormal nodal centrality in both functional and structural networks in the parahippocampal gyrus^19,20,22^. In our results, these decreased connections may result in dysfunction of pain processing and reorganization of the functional networks in female MWoA.

Figures 3 and 4 show that the posterior insula exhibited significantly decreased nodal centrality and the two subregions of the right posterior insula exhibited decreased connectivity with many of brain regions in female MWoA. The insula is a hub region of the brain, and its function is associated with goal-directed cognition, conscious awareness, autonomic regulation, interoception, and somatosensation^31,65^. The insular cortices has been proposed as “a multidimensional integration site for pain”^66^. The posterior insular cortices is connected to the premotor, middle-posterior cingulate, supplementary motor and sensorimotor cortices, indicating its role in sensorimotor integration^28,32–34^, and it has been proposed to play a fundamental role in human pain^35^. The insula has been proposed as a “hub of activity”, as it is connected to frontal, temporal and parietal regions and processes many aspects of complex emotional, cognitive and sensory functions in migraine^31^. Ferot *et al*. investigated the timing of activations across the different sub-regions of insular cortices using intracerebrally recorded nociceptive laser-evoked potentials (LEPs). The results suggested that the posterior insula, which is associated with coding intensity and anatomical location, first processes nociceptive input and then conveys the nociceptive information to the anterior insula, which is related to the processing of the emotional reactions for pain^67^, indicating that the posterior insular cortices is associated with pain perception. Numerous migraine studies have reported that the insula changes structural, functional, and aberrant connections to other regions^10,11,23,26–30,60,68,69^. In addition, we found that the insula was abnormal in right hemisphere. Right-sided lateralization of the insula has also been observed in a number of studies of pain and migraine^24,70,71^. In line with this research, our previous study found that MWoA exhibit significantly decreased cortical thickness in the right insular cortex using surface-based morphometry. It is noteworthy that the volume of posterior insula in female MWoA was smaller compared with healthy controls in current study. Previous findings have suggested that brain function is closely related to anatomical structure^72,73^. Thus, nodal centrality alterations of the insula in female MWoA may be associated with structure changes and the causality of functional and structural changes in posterior insula should be further investigated. In conclusion, female MWoA exhibited decreased nodal centrality and disrupted functional connectivity in the posterior insula, which suggests that female MWoA may exhibit dysfunction of pain processing and perception and that the posterior insula plays an important role in female migraine pathology.

In most previous studies, functional networks were generated at coarse regional level by segmenting the entire brain into 90 sub-regions based on anatomical sulcal information. It has been demonstrated that the different parcellation atlases may result in different topological properties^36,74^. Fan *et al*. designed a human Brainnetome atlas that identified subdivisions of the whole brain (36 subcortical and 210 cortical subregions) based on connectivity parcellation^38^. In this study, we constructed the functional network using a precise connectivity-based parcellation atlas (Brainnetome atlas), and we also compared the differences in topological organizations of networks constructed by the AAL and the Brainnetome atlas. We found that σ and γ of the brain networks of migraineurs were significantly greater than those of controls based on both the AAL and Brainnetome atlas (Fig. 2 and Supplementary Figure S1). However, the nodal centrality of networks based on the AAL atlas showed that many brain regions overlapped with the Brainnetome atlas, including the precentral gyrus, the inferior temporal gyrus, the occipital cortex and the medial prefrontal cortex. In addition, the nodal centrality of networks based on the Brainnetome atlas showed more abnormal brain regions, including the posterior insula, the paracentral lobule, the inferior frontal gyrus, the inferior and superior parietal lobule, the sensory thalamus and the parahippocampus, than the networks based on the AAL atlas. Thus, our results suggest that the different parcellation atlases may result in different topological properties, and the Brainnetome atlas could detect some subtle changes of brain regions and would be complement of structural atlas (e.g. AAL).

The current study sought to investigate the functional network topologic properties of female MWoA based on a precise parcellation atlas (Brainnetome atlas) using graph theory analysis. We found that the topological properties of functional networks in patients were altered compared with controls. The brain regions with changed nodal centrality and disrupted connections were those primarily involved in pain perception, pain modulation or processing and sensory integration processing. Thus, our results may reflect brain network dysfunction or reorganization in female MWoA, which may affect information transmission of pain and pain modulation processing in MWoA. Notably, the posterior insula exhibited decreased nodal centrality, smaller volume and disrupted connectivity with many other brain areas in female migraineurs, which suggests that the posterior insula may be associated with the structural alterations and plays an important role in female migraine pathology. Although all female MWoA were recruited for the current study based strictly on the ICHD-III beta, the clinical heterogeneity of the patient is a primary limitation of this research. Thus, the results of this study should be further validated.

## Electronic supplementary material

Supplementary information
