# Supplementary material for: The Posterior Insula Shows Disrupted Brain Functional Connectivity in Female Migraineurs Without Aura Based on Brainnetome Atlas
Source: Sci Rep. 2017 Dec 4;7:16868. doi: 10.1038/s41598-017-17069-8 (PMC5715029; doi:10.1038/s41598-017-17069-8)

The Posterior Insula Shows Disrupted Brain Functional Connectivity in Female Migraineurs Without Aura Based on Brainnetome Atlas.

Jilei Zhang,[1] Jingjing Su,[2] Mengxing Wang,[1] Ying Zhao,[2] Qi-Ting Zhang,[2] Qian Yao,[2] Haifeng Lu,[1] Hui Zhang,[1] Ge-Fei Li,[2] Yi-Lan Wu,[2] Yi-Sheng Liu,[2] Feng-Di Liu,[2,3] Mei-Ting Zhuang,[2] Yan-Hui Shi,[2, 3] Tian-Yu Hou,[2] Rong Zhao,[2,3] Yuan Qiao,[2,3] Jianqi Li,[1] Jian-Ren Liu,[2,3]*, Xiaoxia Du,[1]*

[1]*Shanghai Key Laboratory of Magnetic Resonance and Department of Physics, School of Physics and Materials Science, East China Normal University, Shanghai 200062, China*

[2]*Department of Neurology and Jiuyuan Municipal Stroke Center, Shanghai Ninth People's Hospital, Shanghai Jiao Tong University School of Medicine, Shanghai 200011, China*

[3]*Clinical Research Center, Shanghai Jiao Tong University School of Medicine, Shanghai 200011, China*

Correspondence and requests for materials should be addressed to X.X.D. (xxdu@phy.ecnu.edu.cn) or J.R.L. (liujr021@vip.163.com)

Supplementary Table S1. The subregions defined by the Brainetome atlas.

Lobe	Gyrus	Left and Right Hemisphere	Label ID.L	Label ID.R	Anatomical and modified Cyto-architectonic descriptions	lh.MNI(X,Y,Z)	rh.MNI(X,Y,Z)
Frontal Lobe	SFG, Superior Frontal Gyrus	SFG_L(R)_7_1	1	2	*A8m, medial area 8*	-5 ,15, 54	7, 16, 54
		SFG_L(R)_7_2	3	4	*A8dl, dorsolateral area 8*	-18, 24, 53	22, 26, 51
		SFG_L(R)_7_3	5	6	*A9l, lateral area 9*	-11, 49, 40	13, 48, 40
		SFG_L(R)_7_4	7	8	*A6dl, dorsolateral area 6*	-18, -1, 65	20, 4, 64
		SFG_L(R)_7_5	9	10	*A6m, medial area 6*	-6, -5, 58	7, -4, 60
		SFG_L(R)_7_6	11	12	*A9m,medial area 9*	-5, 36, 38	6, 38, 35
		SFG_L(R)_7_7	13	14	*A10m, medial area 10*	-8, 56, 15	8, 58, 13
	MFG, Middle Frontal Gyrus	MFG_L(R)_7_1	15	16	*A9/46d, dorsal area 9/46*	-27, 43, 31	30, 37, 36
		MFG_L(R)_7_2	17	18	*IFJ, inferior frontal junction*	-42, 13, 36	42, 11, 39
		MFG_L(R)_7_3	19	20	*A46, area 46*	-28, 56, 12	28, 55, 17
		MFG_L(R)_7_4	21	22	*A9/46v, ventral area 9/46*	-41, 41, 16	42, 44, 14
		MFG_L(R)_7_5	23	24	*A8vl, ventrolateral area 8*	-33, 23, 45	42, 27, 39
		MFG_L(R)_7_6	25	26	*A6vl, ventrolateral area 6*	-32, 4, 55	34, 8, 54

	MFG_L(R)_7_7	27	28	*A10l, lateral area10*	-26, 60, -6	25, 61, -4
IFG, Inferior Frontal Gyrus	IFG_L(R)_6_1	29	30	*A44d,dorsal area 44*	-46, 13, 24	45, 16, 25
	IFG_L(R)_6_2	31	32	*IFS, inferior frontal sulcus*	-47, 32, 14	48, 35, 13
	IFG_L(R)_6_3	33	34	*A45c, caudal area 45*	-53, 23, 11	54, 24, 12
	IFG_L(R)_6_4	35	36	*A45r, rostral area 45*	-49, 36, -3	51, 36, -1
	IFG_L(R)_6_5	37	38	*A44op, opercular area 44*	-39, 23, 4	42, 22, 3
	IFG_L(R)_6_6	39	40	*A44v, ventral area 44*	-52, 13, 6	54, 14, 11
OrG, Orbital Gyrus	OrG_L(R)_6_1	41	42	*A14m, medial area 14*	-7, 54, -7	6, 47, -7
	OrG_L(R)_6_2	43	44	*A12/47o, orbital area 12/47*	-36, 33, -16	40, 39, -14
	OrG_L(R)_6_3	45	46	*A11l, lateral area 11*	-23, 38, -18	23, 36, -18
	OrG_L(R)_6_4	47	48	*A11m, medial area 11*	-6, 52, -19	6, 57, -16
	OrG_L(R)_6_5	49	50	*A13, area 13*	-10, 18, -19	9, 20, -19
	OrG_L(R)_6_6	51	52	*A12/47l, lateral area 12/47*	-41, 32, -9	42, 31, -9
PrG, Precentral Gyrus	PrG_L(R)_6_1	53	54	*A4hf, area 4(head and face region)*	-49, -8, 39	55, -2, 33
	PrG_L(R)_6_2	55	56	*A6cdl, caudal dorsolateral area 6*	-32, -9, 58	33, -7, 57

		PrG_L(R)_6_3	57	58	*A4ul, area 4(upper limb region)*	-26, -25, 63	34, -19, 59
		PrG_L(R)_6_4	59	60	*A4t, area 4(trunk region)*	-13, -20, 73	15, -22, 71
		PrG_L(R)_6_5	61	62	*A4tl, area 4(tongue and larynx region)*	-52, 0, 8	54, 4, 9
		PrG_L(R)_6_6	63	64	*A6cvl, caudal ventrolateral area 6*	-49, 5, 30	51, 7, 30
	PCL, Paracentral Lobule	PCL_L(R)_2_1	65	66	*A1/2/3ll, area1/2/3 (lower limb region)*	-8, -38, 58	10, -34, 54
		PCL_L(R)_2_2	67	68	*A4ll, area 4, (lower limb region)*	-4, -23, 61	5, -21, 61
Temporal Lobe	STG, Superior Temporal Gyrus	STG_L(R)_6_1	69	70	*A38m, medial area 38*	-32, 14, -34	31, 15, -34
		STG_L(R)_6_2	71	72	*A41/42, area 41/42*	-54, -32, 12	54, -24, 11
		STG_L(R)_6_3	73	74	*TE1.0 and TE1.2*	-50, -11, 1	51, -4, -1
		STG_L(R)_6_4	75	76	*A22c, caudal area 22*	-62, -33, 7	66, -20, 6
		STG_L(R)_6_5	77	78	*A38l, lateral area 38*	-45, 11, -20	47, 12, -20
		STG_L(R)_6_6	79	80	*A22r, rostral area 22*	-55, -3, -10	56, -12, -5
	MTG, Middle Temporal Gyrus	MTG_L(R)_4_1	81	82	*A21c, caudal area 21*	-65, -30, -12	65, -29, -13
		MTG_L(R)_4_2	83	84	*A21r, rostral area 21*	-53, 2, -30	51, 6, -32

	MTG_L(R)_4_3	85	86	*A37dl, dorsolateral area37*	-59, -58, 4	60, -53, 3
	MTG_L(R)_4_4	87	88	*aSTS, anterior superior temporal sulcus*	-58, -20, -9	58, -16, -10
ITG, Inferior Temporal Gyrus	ITG_L(R)_7_1	89	90	*A20iv, intermediate ventral area 20*	-45, -26, -27	46, -14, -33
	ITG_L(R)_7_2	91	92	*A37elv, extreme lateroventral area37*	-51, -57, -15	53, -52, -18
	ITG_L(R)_7_3	93	94	*A20r, rostral area 20*	-43, -2, -41	40, 0, -43
	ITG_L(R)_7_4	95	96	*A20il, intermediate lateral area 20*	-56, -16, -28	55, -11, -32
	ITG_L(R)_7_5	97	98	*A37vl, ventrolateral area 37*	-55, -60, -6	54, -57, -8
	ITG_L(R)_7_6	99	100	*A20cl, caudolateral of area 20*	-59, -42, -16	61, -40, -17
	ITG_L(R)_7_7	101	102	*A20cv, caudoventral of area 20*	-55, -31, -27	54, -31, -26
FuG, Fusiform Gyrus	FuG_L(R)_3_1	103	104	*A20rv, rostroventral area 20*	-33, -16, -32	33, -15, -34
	FuG_L(R)_3_2	105	106	*A37mv, medioventral area37*	-31, -64, -14	31, -62, -14
	FuG_L(R)_3_3	107	108	*A37lv, lateroventral area37*	-42, -51, -17	43, -49, -19
PhG, Parahippocampal Gyrus	PhG_L(R)_6_1	109	110	*A35/36r, rostral area 35/36*	-27, -7, -34	28, -8, -33
	PhG_L(R)_6_2	111	112	*A35/36c, caudal area 35/36*	-25, -25, -26	26, -23, -27

		PhG_L(R)_6_3	113	114	*TL, area TL (lateral PPHC, posterior parahippocampal gyrus)*	-28, -32, -18	30, -30, -18
		PhG_L(R)_6_4	115	116	*A28/34, area 28/34 (EC, entorhinal cortex)*	-19, -12, -30	19, -10, -30
		PhG_L(R)_6_5	117	118	*TI, area TI(temporal agranular insular cortex)*	-23, 2, -32	22, 1, -36
		PhG_L(R)_6_6	119	120	*TH, area TH (medial PPHC)*	-17, -39, -10	19, -36, -11
	pSTS, posterior Superior Temporal Sulcus	pSTS_L(R)_2_1	121	122	*rpSTS, rostroposterior superior temporal sulcus*	-54, -40, 4	53, -37, 3
		pSTS_L(R)_2_2	123	124	*cpSTS, caudoposterior superior temporal sulcus*	-52, -50, 11	57, -40, 12
Parietal Lobe	SPL, Superior Parietal Lobule	SPL_L(R)_5_1	125	126	*A7r, rostral area 7*	-16, -60, 63	19, -57, 65
		SPL_L(R)_5_2	127	128	*A7c, caudal area 7*	-15, -71, 52	19, -69, 54
		SPL_L(R)_5_3	129	130	*A5l, lateral area 5*	-33, -47, 50	35, -42, 54
		SPL_L(R)_5_4	131	132	*A7pc, postcentral area 7*	-22, -47, 65	23, -43, 67
		SPL_L(R)_5_5	133	134	*A7ip, intraparietal area 7(hIP3)*	-27, -59, 54	31, -54, 53
		IPL_L(R)_6_1	135	136	*A39c, caudal area 39(PGp)*	-34, -80, 29	45, -71, 20

	IPL, Inferior Parietal Lobule	IPL_L(R)_6_2	137	138	*A39rd, rostrodorsal area 39(Hip3)*	-38, -61, 46	39, -65, 44
		IPL_L(R)_6_3	139	140	*A40rd, rostrodorsal area 40(PFt)*	-51, -33, 42	47, -35, 45
		IPL_L(R)_6_4	141	142	*A40c, caudal area 40(PFm)*	-56, -49, 38	57, -44, 38
		IPL_L(R)_6_5	143	144	*A39rv, rostroventral area 39(PGa)*	-47, -65, 26	53, -54, 25
		IPL_L(R)_6_6	145	146	*A40rv, rostroventral area 40(PFop)*	-53, -31, 23	55, -26, 26
	Pcun, Precuneus	PCun_L(R)_4_1	147	148	*A7m, medial area 7(PEp)*	-5, -63, 51	6, -65, 51
		PCun_L(R)_4_2	149	150	*A5m, medial area 5(PEm)*	-8, -47, 57	7, -47, 58
		PCun_L(R)_4_3	151	152	*dmPOS, dorsomedial parietooccipital sulcus(PEr)*	-12, -67, 25	16, -64, 25
		PCun_L(R)_4_4	153	154	*A31, area 31 (Lc1)*	-6, -55, 34	6, -54, 35
	PoG, Postcentral Gyrus	PoG_L(R)_4_1	155	156	*A1/2/3ulhf, area 1/2/3(upper limb, head and face region)*	-50, -16, 43	50, -14, 44
		PoG_L(R)_4_2	157	158	*A1/2/3tonIa, area 1/2/3(tongue and larynx region)*	-56, -14, 16	56, -10, 15
		PoG_L(R)_4_3	159	160	*A2, area 2*	-46, -30, 50	48, -24, 48
		PoG_L(R)_4_4	161	162	*A1/2/3tru, area1/2/3(trunk region)*	-21, -35, 68	20, -33, 69

Insular Lobe	INS, Insular Gyrus	INS_L(R)_6_1	163	164	*G, hypergranular insula*	-36, -20, 10	37, -18, 8
		INS_L(R)_6_2	165	166	*vIa, ventral agranular insula*	-32, 14, -13	33, 14, -13
		INS_L(R)_6_3	167	168	*dIa, dorsal agranular insula*	-34, 18, 1	36, 18, 1
		INS_L(R)_6_4	169	170	*vId/vIg, ventral dysgranular and granular insula*	-38, -4, -9	39, -2, -9
		INS_L(R)_6_5	171	172	*dIg, dorsal granular insula*	-38, -8, 8	39, -7, 8
		INS_L(R)_6_6	173	174	*dId, dorsal dysgranular insula*	-38, 5, 5	38, 5, 5
Limbic Lobe	CG, Cingulate Gyrus	CG_L(R)_7_1	175	176	*A23d, dorsal area 23*	-4, -39, 31	4, -37, 32
		CG_L(R)_7_2	177	178	*A24rv, rostroventral area 24*	-3, 8, 25	5, 22, 12
		CG_L(R)_7_3	179	180	*A32p, pregenual area 32*	-6, 34, 21	5, 28, 27
		CG_L(R)_7_4	181	182	*A23v, ventral area 23*	-8, -47, 10	9, -44, 11
		CG_L(R)_7_5	183	184	*A24cd, caudodorsal area 24*	-5, 7, 37	4, 6, 38
		CG_L(R)_7_6	185	186	*A23c, caudal area 23*	-7, -23, 41	6, -20, 40
		CG_L(R)_7_7	187	188	*A32sg, subgenual area 32*	-4, 39, -2	5, 41, 6
		MVOcC _L(R)_5_1	189	190	*cLinG, caudal lingual gyrus*	-11, -82, -11	10, -85, -9

Occipital Lobe	MVOcC, MedioVentral Occipital Cortex	MVOcC _L(R)_5_2	191	192	*rCunG, rostral cuneus gyrus*	-5, -81, 10	7, -76, 11
		MVOcC _L(R)_5_3	193	194	*cCunG, caudal cuneus gyrus*	-6, -94, 1	8, -90, 12
		MVOcC _L(R)_5_4	195	196	*rLinG, rostral lingual gyrus*	-17, -60, -6	18, -60, -7
		MVOcC _L(R)_5_5	197	198	*vmPOS,ventromedial parietooccipital sulcus*	-13, -68, 12	15, -63, 12
	LOcC, lateral Occipital Cortex	LOcC_L(R)_4_1	199	200	*mOccG, middle occipital gyrus*	-31, -89, 11	34, -86, 11
		LOcC _L(R)_4_2	201	202	*V5/MT+, area V5/MT+*	-46, -74, 3	48, -70, -1
		LOcC _L(R)_4_3	203	204	*OPC, occipital polar cortex*	-18, -99, 2	22, -97, 4
		LOcC_L(R)_4_4	205	206	*iOccG, inferior occipital gyrus*	-30, -88, -12	32, -85, -12
		LOcC _L(R)_2_1	207	208	*msOccG, medial superior occipital gyrus*	-11, -88, 31	16, -85, 34
		LOcC _L(R)_2_2	209	210	*lsOccG, lateral superior occipital gyrus*	-22, -77, 36	29, -75, 36
Subcortical Nuclei	Amyg, Amygdala	Amyg_L(R)_2_1	211	212	*mAmyg, medial amygdala*	-19, -2, -20	19, -2, -19
		Amyg_L(R)_2_2	213	214	*lAmyg, lateral amygdala*	-27, -4, -20	28, -3, -20
	Hipp, Hippocampus	Hipp_L(R)_2_1	215	216	*rHipp, rostral hippocampus*	-22, -14, -19	22, -12, -20

	Hipp_L(R)_2_2	217	218	*cHipp, caudal hippocampus*	-28, -30, -10	29, -27, -10
BG, Basal Ganglia	BG_L(R)_6_1	219	220	*vCa, ventral caudate*	-12, 14, 0	15, 14, -2
	BG_L(R)_6_2	221	222	*GP, globus pallidus*	-22, -2, 4	22, -2, 3
	BG_L(R)_6_3	223	224	*NAC, nucleus accumbens*	-17, 3, -9	15, 8, -9
	BG_L(R)_6_4	225	226	*vmPu, ventromedial putamen*	-23, 7, -4	22, 8, -1
	BG_L(R)_6_5	227	228	*dCa, dorsal caudate*	-14, 2, 16	14, 5, 14
	BG_L(R)_6_6	229	230	*dlPu, dorsolateral putamen*	-28, -5, 2	29, -3, 1
Tha, Thalamus	Tha_L(R)_8_1	231	232	*mPFtha, medial pre-frontal thalamus*	-7, -12, 5	7, -11, 6
	Tha_L(R)_8_2	233	234	*mPMtha, pre-motor thalamus*	-18, -13, 3	12, -14, 1
	Tha_L(R)_8_3	235	236	*Stha, sensory thalamus*	-18, -23, 4	18, -22, 3
	Tha_L(R)_8_4	237	238	*rTtha, rostral temporal thalamus*	-7, -14, 7	3, -13, 5
	Tha_L(R)_8_5	239	240	*PPtha, posterior parietal thalamus*	-16, -24, 6	15, -25, 6
	Tha_L(R)_8_6	241	242	*Otha, occipital thalamus*	-15, -28, 4	13, -27, 8
	Tha_L(R)_8_7	243	244	*cTtha, caudal temporal thalamus*	-12, -22, 13	10, -14, 14

	Tha_L(R)_8_8	245	246	*lPFtha, lateral pre-frontal thalamus*	-11, -14, 2	13, -16, 7

Supplementary Table S2. The definitions of network measures.

Measures	Definitions
small-world parameters	
L_p<br>characteristic path length	The average of all nodal L_i. The formula is following:<br>$$L_p = \frac{1}{n}\sum_{j \in N} L_i,\ L_i = \frac{1}{n-1}\sum_{j\in N, j\neq i} d_{ij}$$<br>Where L_i is the average distance between node *i* and all other nodes.
C_p<br>clustering coefficient	The average of all nodal C_i. The formula is following:<br>$$C_p = \frac{1}{n}\sum_{j \in N} C_i,$$<br>Where C_i is the clustering coefficient of node *i*.
λ<br>normalized characteristic path length	The λ is the ratio of L_p and the same metrics estimated in random networks. The formula is following:<br>$$\lambda = \frac{L_p}{L_{p(rand)}}$$
γ<br>normalized clustering coefficient	The γ is the ratio of L_p and the same metrics estimated in random networks. The formula is following:<br>$$\gamma = \frac{C_p}{C_{p(rand)}}$$
σ<br>small-worldness	The σ is the ratio of γ and λ. The formula is following:<br>$$\sigma = \frac{\gamma}{\lambda}$$<br>Small-world networks often have $\sigma \gg 1$
network efficiency	
E_{glob}<br>global efficiency	The average of all node E_i. The formula is following:<br>$$E_{glob} = \frac{1}{n}\sum_{j \in N} E_{i,}\quad E_i = \frac{1}{n-1}\sum_{j \in N, j\neq i}\frac{1}{d_{ij}}$$

	where E_i is the efficiency of node *i*.
E_{loc}<br>local efficiency	The average of all node $E_{loc,i.}$ The formula is following:<br>$$E_{loc} = \frac{1}{n}\sum_{j \in N} E_{loc,i},$$<br>where $E_{loc,i}$ is the local efficiency of node *i*.
nodal centrality	
Betweenness	The fraction of all shortest paths in the network that pass through node i. The formula is following:<br>$$B_i = \frac{1}{(n-1)(n-2)} \sum_{\substack{h,j \in N, \\ h \neq j, h \neq = i, j \neq i}} \frac{P_{hj}(i)}{P_{hj}}$$<br>where P_{hj} is the number of shortest paths between h and j, and $P_{hj}(i)$ is the number of shortest paths between h and j that pass through i.
Efficiency	The efficiency between node i and all other nodes. The formula is following:<br>$$E_i = \frac{1}{n-1} \sum_{j \in N, j \neq i} \frac{1}{d_{ij}}$$
Degree	The number of links connected to node *i.* The formula is following:<br>$$k_i = \sum_{j \in N} a_{ij}$$

N is the set of all nodes and n is the number of nodes in a given network, a_{ij} is the connection status between i and j. d_{ij} is the shortest path length between node i and j. The GRETNA toolbox contains functions to compute all measures in this table.

Supplementary Table S3. Regions of brain within AAL atlas showing abnormal nodal centrality in female MWoA versus healthy controls.

Brain regions	Anatomical Structure	Nodal betweenness Mean(SD)			Nodal efficiency Mean(SD)			Nodal degree Mean(SD)		
		FMWoA	HC	P value	FMWoA	HC	P value	FMWoA	HC	P value
Nodal centrality increase in migraineurs										
ORBmid.R	Middle frontal gyrus, orbital part	3.59(3.38)	4.81(7.93)	0.232	**0.094(0.016)**	**0.078(0.026)**	**0.004**	2.28(1.13)	1.52(1.27)	0.010
SFGmed.L	Superior frontal gyrus, medial	10.40(7.66)	10.01(10.73)	0.437	0.110(0.015)	0.100(0.014)	0.006	**4.03(1.60)**	**2.88(1.46)**	**0.003**
SFGmed.R	Superior frontal gyrus, medial	9.35(7.89)	7.55(7.30)	0.190	**0.109(0.014)**	**0.097(0.012)**	**0.000***	**3.98(1.55)**	**2.54(1.27)**	**0.000***
PCG.R	Posterior cingulate gyrus	4.11(5.52)	3.00(4.80)	0.214	**0.094(0.015)**	**0.078(0.028)**	**0.004**	2.25(1.28)	1.51(1.49)	0.022
ITG.L	Inferior temporal gyrus	19.67(16.01)	11.75(10.85)	0.151	0.118(0.013)	0.106(0.016)	**0.001***	**5.06(1.71)**	**3.48(1.71)**	**0.000***
Nodal centrality decrease in migraineurs										
PreCG.L	Precental gyrus	10.21(8.26)	14.87(11.20)	0.038	**0.107(0.019)**	**0.119(0.012)**	**0.001***	**3.82(1.83)**	**5.46(1.62)**	**0.000***

ORBmid.L	Middle frontal gyrus, orbital part	**7.79(6.23)**	**17.51(15.88)**	**0.000**	0.101(0.013)	0.093(0.022)	0.051	2.95(1.17)	2.63(1.68)	0.201
SOG.L	Superior occipital gyrus	**5.33(6.29)**	6.63(5.70)	0.213	**0.107(0.012)**	**0.116(0.008)**	**0.002***	**4.01(1.45)**	**4.98(1.22)**	**0.003**
MOG.L	Middle occipital gyrus	16.43(15.34)	17.32(10.34)	0.401	0.117(0.013)	0.125(0.010)	0.006	**5.03(1.56)**	**6.18(1.59)**	**0.003**
PoCG.L	Postcentral gyrus	7.70(8.31)	9.91(8.23)	0.158	**0.107(0.015)**	**0.117(0.011)**	**0.003**	**3.98(1.77)**	**5.23(1.56)**	**0.003**
PAL.L	Lenticular nucleus, pallidum	**2.89(3.47)**	**7.53(8.00)**	**0.002**	0.082(0.020)	0.081(0.024)	0.449	1.75(1.02)	1.86(1.35)	0.362

Brain areas were considered abnormal in female MWoA if they exhibited signifcant between-group differences (50000 permutations, P< 0.005 shown in bold font) in at least one of the three nodal centralities. "*" indicates brain regions that are significant after multiple comparison correction (P<0.05, nonparametric permutation test, FDR corrected). SD: Standard Deviation.

Supplementary Figure S1. The differences in the topological properties of functional networks based on the AAL atlas between female MWoA and healthy controls. Error bars denote standard deviations. *: significant difference between two groups (P<0.05, 50000 permutations test, uncorrected); HC: healthy controls; FM: female migraineurs without aura; E_{glob}: global efficiency; E_{loc}: local efficiency; C_p: clustering coefficient; γ: normalized clustering coefficient; λ: normalized characteristic path length; L_p: characteristic path length; σ: small-worldness.

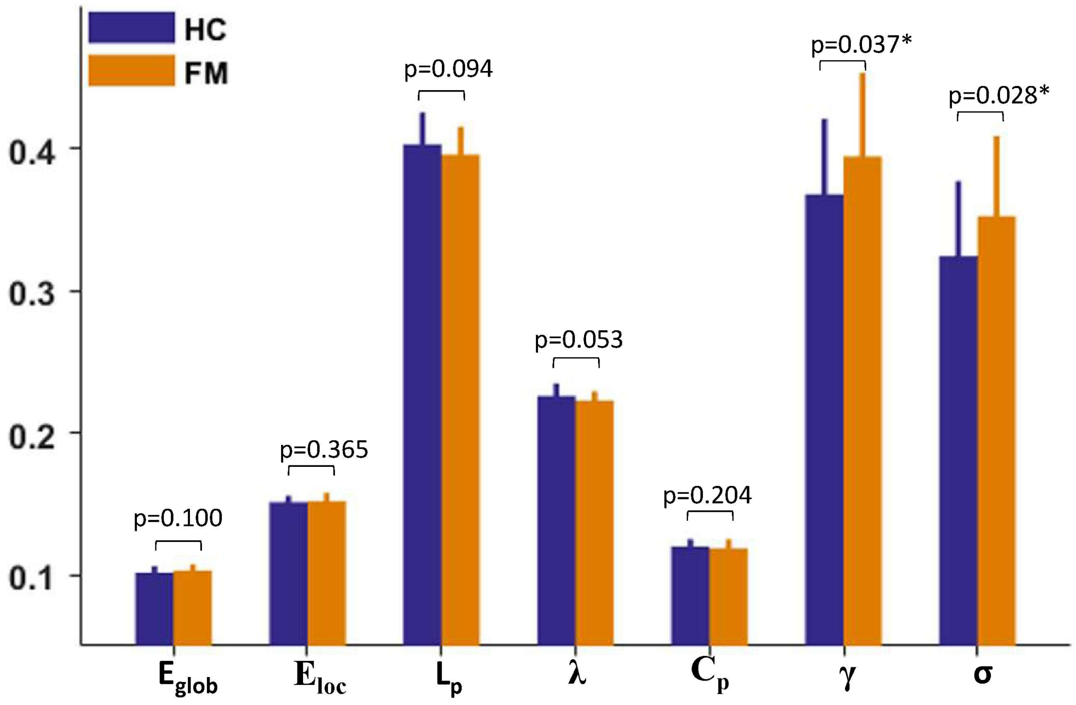

Supplementary Figure S2. Brain regions within AAL atlas showing abnormal nodal centrality in female MWoA comparing with healthy controls. The nodal centrality of female MWO exhibited significantly increased (red color) in the bilateral medial prefrontal cortex, the right orbital frontal cortex, the posterior cingulate gyrus and the left inferior temporal gyrus and decreased (blue color) in the left precentral gyrus, the left orbital frontal gyrus, the left superior and the middle occipital gyrus, the left postcentral gyrus, and the left pallidum.

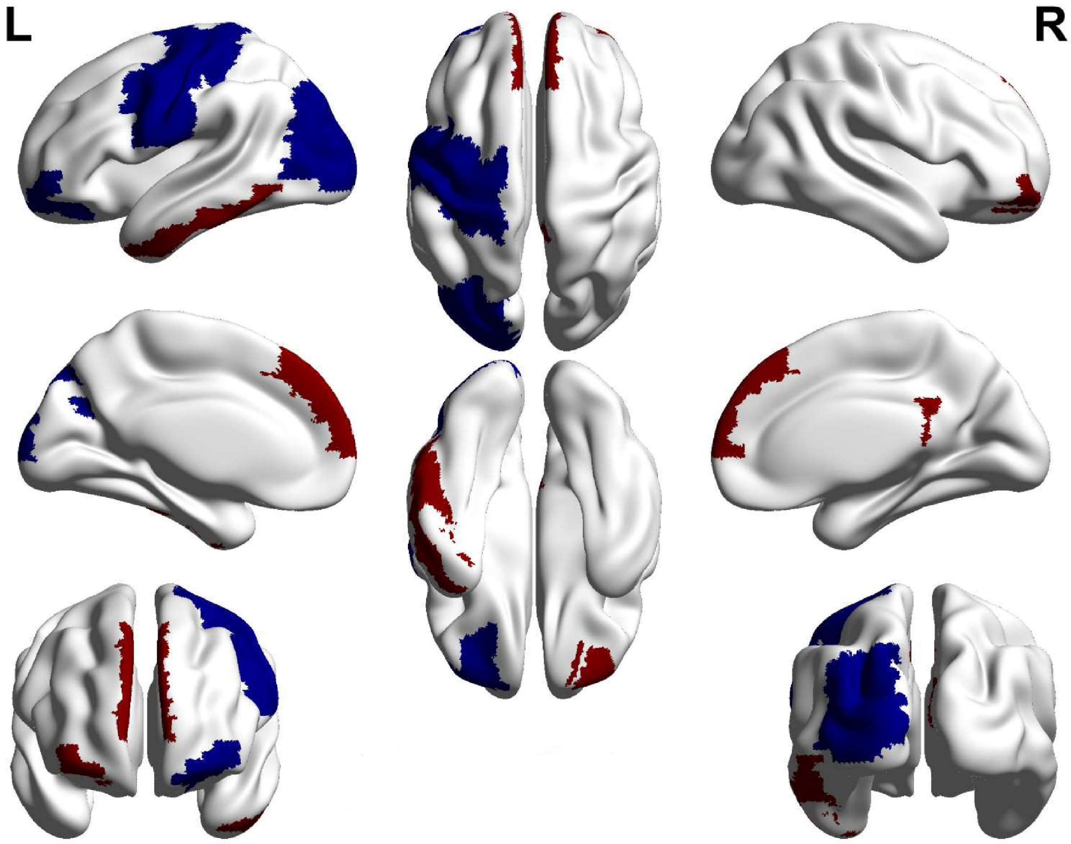

Supplement: Supplementary file 1 — Supplementary information [file 41598_2017_17069_MOESM1_ESM.pdf]
